# Structured Development of Learning and Assessment Tasks to Prevent Generative AI Misuse and Enhance AI Literacy in the Faculty in Physiotherapy Education

**DOI:** 10.1177/23821205251378794

**Published:** 2025-09-16

**Authors:** Yvonne Lindbäck, Karin Valeskog, Karin Schröder, Sofi Sonesson

**Affiliations:** 1 Unit of Physiotherapy, Department of Health, Medicine and Caring Sciences, 4566 Linköping University, Linköping, Sweden

**Keywords:** faculty development, detection and prevention, plagiarism, AI literacy, clinical reasoning

## Abstract

**Objective:**

The rapid emergence of generative artificial intelligence (GAI) in higher education necessitates redesign of learning activities and assessments to uphold academic integrity and foster AI literacy. This article presents a structured approach to developing educational strategies that mitigate GAI misuse while enhancing students’ understanding of GAI, with a focus on collaborative faculty engagement and curricular adaptation in physiotherapy education.

**Methods:**

Using the Quality Implementation Framework (QIF), we conducted a comprehensive review of all courses within a Swedish physiotherapy program employing a problem-based learning (PBL) model. Faculty-wide, time-bound development initiatives were implemented, including targeted AI literacy training. A student survey was conducted to assess GAI usage patterns and perceptions.

**Results:**

Assessment formats were adapted to emphasize clinical reasoning and critical thinking, reducing opportunities for GAI misuse. Standardized guidelines on acceptable GAI use were integrated across all courses. The survey results 2 months after implementation indicated diverse usage patterns: 13% of students reported daily use of GAI, while 24% had never used it. Additionally, 42% felt adequately informed about GAI. Faculty AI literacy and confidence improved through structured group work and feedback, supporting the integration of AI-related tasks into the curriculum.

**Conclusions:**

The systematic approach using QIF and PBL, expert support, faculty champions, problem-solving strategies, and feedback, enabled meaningful curricular changes within 4 months. The variability in student GAI use underscores the need for equitable AI literacy education. This approach not only reduced the risk of GAI misuse but also enhanced faculty preparedness, offering a scalable model for other health sciences programs.

## Introduction

Since the launch of ChatGPT by Open AI in November 2022, generative artificial intelligence (GAI) has rapidly advanced and become increasingly integrated into higher education. Despite its widespread adoption and enhanced capabilities, understanding of GAI among teachers and students remains limited. AI literacy, which encompasses the ability to critically evaluate AI technologies, communicate and collaborate with AI, and effectively utilize AI in various contexts,^1^ is now a crucial competency. However, fostering these skills presents a significant pedagogical challenge.

GAI offers several advantages for students, including easy access to information and assistance in summarizing and enhancing texts. Yet, current plagiarism detection tools often fail to identify AI-generated text.^2^ A study found that chatbot-generated responses in take-home exams received pass rates ranging from 38% to 86%, while student-written texts were frequently downgraded. It was also concluded that the presence of AI chatbots may reduce teachers confidence in students’ texts, requiring them to be more thorough and suspicious when assessing a text.^3^ Additionally, teachers have expressed concerns about fairness, accountability, and a lack of institutional support or resources for integrating GAI into teaching.^4^ We have previously reported that physiotherapy students acknowledge the benefits of GAI, but also express concerns about its impact on learning quality. They emphasized the importance of a critical approach and institutional guidance regarding acceptable GAI use.^5^

A core learning objective in physiotherapy education is the development of clinical reasoning (CR) to enable informed decisions and person-centered actions.^6^ Strengthening CR is crucial for reducing diagnostic errors and improving timely diagnoses.^7^ Best practices in teaching, learning, and assessment are vital to support this skill.^8^ Overreliance on GAI in medical education poses a risk of declining critical thinking and clinical decision-making skills.^9^ Furthermore, although ChatGPT-generated responses to CR questions may appear comprehensive, they frequently lack causal reasoning and demonstrate limited cognitive depth. It is essential for students to recognize these limitations and develop more effective strategies for engaging with GAI, including refining their prompting techniques.^10^ This highlights the need to revise pedagogical approaches and assessment designs to ensure they continue to support learning objectives, particularly in CR and critical thinking.^9^

In the Faculty of Medicine and Health Sciences, students are likely to encounter GAI in future professional roles—as users, translators, or even developers of AI tools. This necessitates competencies in technical understanding, validation, critical appraisal, and ethical considerations.^11^ Consequently, it is recommended that curricula explicitly address these areas, establish clear policies for GAI use to protect institutional and patient data, and promote interdisciplinary collaboration.^12^

This article presents a structured approach to developing educational strategies that mitigate GAI misuse while enhancing students’ understanding of AI, with a focus on collaborative faculty engagement and curricular adaptation in physiotherapy education. The quality implementation framework (QIF)^13^ was applied to structure the development initiatives.

## Methods

The setting for this GAI initiative was a Swedish physiotherapy program, using problem-based learning (PBL) which is a student-centered pedagogical approach, where the students learn by analyzing authentic problems.^14,15^ Typically conducted in small groups, PBL is facilitated by a tutor who guides the learning process rather than delivering direct instruction. This method facilitates the development of domain-specific knowledge, professional competence, collaboration, problem-solving abilities, critical thinking, and independent learning. Through active inquiry and peer interaction, students are encouraged to take ownership of their learning, reflect on their reasoning, and apply theoretical concepts to practical scenarios.^16^

**Figure 1.**
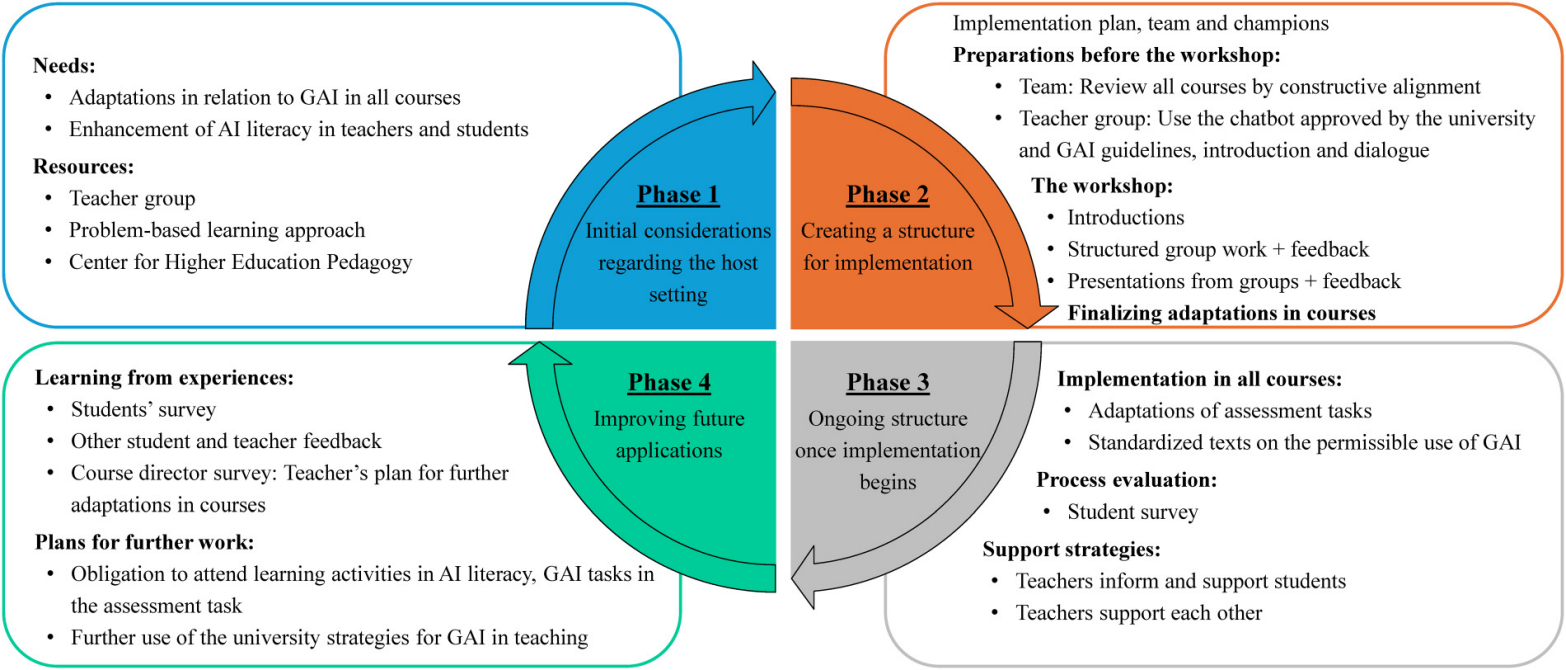
Implementation Activities Across the 4 Quality Implementation Framework (QIF) Phases.^13^

The implementation of development activities was structured according to the QIF developed by Meyers et al^13^ which outlines 4 key phases for successful educational change (1) initial considerations regarding the host settings, (2) creating a structure for implementation, (3) ongoing structure once implementation begins, and (4) improving future applications (Figure 1). The reporting of this study conforms to the Standards for QUality Improvement Reporting Excellence in Education statement (SQUIRE-EUD)^17^ (see Supplemental Material).

### QIF Phase 1: Initial Considerations

The first task was to assess institutional and pedagogical needs related to GAI. Our university's guidelines emphasize that faculty must understand GAI's pedagogical applications and ensure legally sound assessment practices. For students, the guideline encourages GAI usage to support understanding and learning, while clarifying their responsibility to know permitted use. Unauthorized GAI usage is considered academic misconduct and must be reported to the Disciplinary Board. These policies underscored the need to strengthen AI literacy among faculty. In this phase, some faculty members expressed the view that AI literacy should be addressed by external resources, as it was perceived to fall outside the scope of their teaching responsibilities. Enhancing faculty AI literacy was therefore a key objective throughout the implementation process. The aim was to ensure that all teachers felt confident in disseminating information and engaging in dialogue with students about the use of GAI tools.

The next task was to evaluate our readiness and ability to provide sufficient support for the implementation. Given the faculty's experience with the PBL approach^14,15^ we incorporated problem-solving strategies, group work with presentations, and feedback into the initiative. These elements align with PBL principles^16^ and promote critical engagement with GAI. Additional support was provided by the university's Center for Higher Education Pedagogy, which contributed expertise in GAI-enhanced teaching.

### QIF Phase 2: Creating a Structure for Implementation

**Figure 2.**
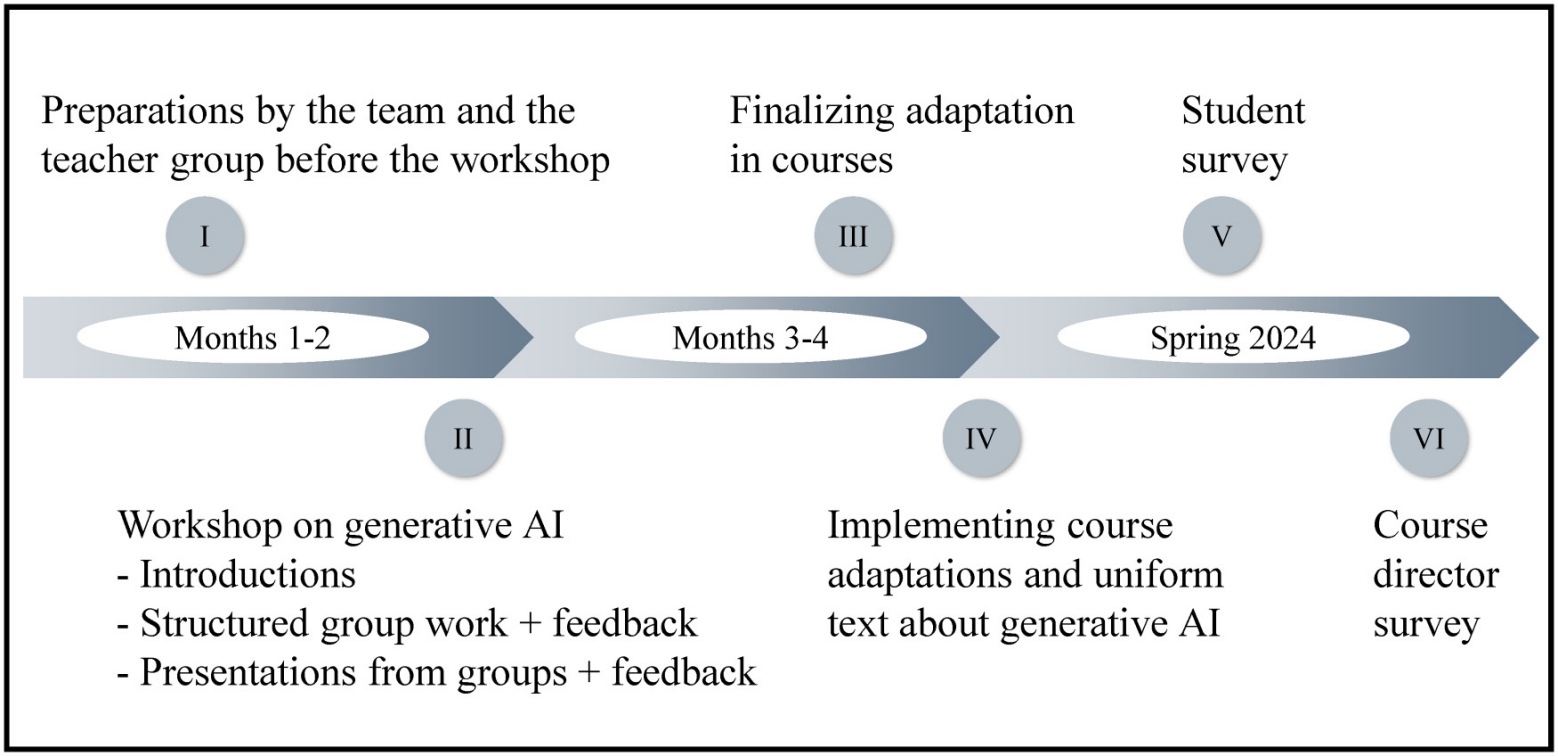
The 4-Month Implementation Plan for Adapting Learning Activities and Assessment Tasks to Mitigate the Misuse of Generative Artificial Intelligence (GAI) and to Enhance AI Literacy.

To effectively support implementation, we formed a team^18^ including the head of the division (team leader), the program director, the subject representative, an educational developer, 2 course directors who served as clinical champions,^19^ and support from the Center for Higer Education Pedagogy. A team-based approach is associated with improved outcomes^20^ and is better suited to understand and address end-user needs, offering diverse perspectives.^20,21^ The implementation team developed the time-bound to 4-month implementation plan (Figure 2).

The first 2 months focused on preparation work ahead of a 2-day faculty workshop on GAI. The initial task involved the head of the division and 3 team members reviewing all courses and assessing risks of GAI misuse in assessment tasks. This review was structured using a constructive alignment matrix,^22^ which links intended learning outcomes, teaching and learning activities, and assessment tasks. Each course's digital matrix facilitated the identification of misalignments and vulnerabilities where GAI use could undermine learning. These findings informed the development of a standardized form for group work and presentations, covering risk verification, assessment adjustments, criteria, required resources, anticipated benefits, potential risks, and curriculum changes. The clinical champions, addressed implementation barriers^19^ and prepared course-specific presentations for the workshop. To build foundational knowledge, teachers’ tasks were to create accounts with a university-approved chatbot (free for faculties and students) and explore its capabilities. This preceded a lecture and dialogue session led by an expert from the Center for Higher Education Pedagogy, aimed at enhancing GAI understanding.

The workshop started with an overview of the university's GAI guidelines, emphasizing that while GAI may support brainstorming and learning, students must remain the authors of their submitted work. In cases where GAI use is not allowed, assessment tasks can be conducted in controlled environments without internet access. The guidelines also include 7 strategies to prevent GAI misuse, tailored to the subject, intended learning outcomes, and assessment task design (Table 1). The clinical champions presented their course reviews and adaptations as models for the faculty. The faculty then worked in groups to review assessment tasks prone to GAI misuse, complete forms detailing the risks for each task and present their proposed adaptations for feedback. A default text on GAI use was developed for course syllabi, adaptable to specific assessment contexts.

**Table 1.** Educational Strategies and Examples of Adaptations to Assessment Tasks to Reduce the Risk of Misuse of GAI and Ensure Accurate Evaluation of Students’ Knowledge While Enhancing AI Literacy.

Educational strategies to prevent misuse of GAI*	Examples of adaptations to assessment tasks
1. Focus on the process, not just the product	Use of a stepwise process for written assignments, involving teachers in some checkpoints, or using peer review as a TLA. For certain assignments, students need to document their revisions, explain the reasons for their revisions, and whether the revisions were based on GAI or peer feedback
2. Include personal experiences	Require written assignments to be anchored to personal patient cases to apply new knowledge and use clinical reasoning
3. Include empirical data	Require submission of a screenshot of the search history (database, search query, used filters, search result, etc)^
4. Include course details	Recommend incorporating course details from for example lectures and patient cases in written assignments
5. Use different modalities	Combine written assignments with other modalities, such as answering questions in a seminar based on the written text or applying knowledge from the text in a new context. Include an audio file in the submission consisting of a lecture based on the text but in this lecture applying new knowledge in a case scenario to challenge clinical reasoning
6. Change the form of the examination	Prepare for oral examination by planning an examination, assessment, and treatment based on a patient case, without access to the internet and books. This is made to preserve preparation using clinical reasoning
7. Use of GAI	Mandate the use of GAI for feedback in a written assignment. Students should write a short reflection on what feedback was used, how, and why. These reflections are discussed in a seminar to critically assess feedback from GAI and hopefully support writing skills^
8. Preserve or improve the assessment of clinical reasoning and critical thinking	In seminars and group discussions, students need to elaborate on their clinical reasoning to a greater extent, to attain ILO Changes in instructions with more focus on reasoning in the text about different studies and synthesizing, rather than describing and summarizing individual studies, to enhance critical thinking
9. Enhance AI literacy	Make it mandatory to watch a film produced by the university library on what GAI is and how it can be used to support students’ learning^

* Strategies 1 to 7 are the University's 7 educational strategies to prevent misuse of GAI and strategies 8 and 9 were produced as part of this development initiative.

^Plans to be implemented.

Abbreviations: GAI, generative artificial intelligence; ILO, intended learning outcomes; TLA, teaching and learning activity.

Following the workshop, the work groups’ tasks were to finalize course-specific adaptations. The team leader and the educational developer had status update meetings with faculty members, and the process was subsequently followed up in unit meetings to facilitate knowledge sharing and ensure alignment across the curriculum. A central focus throughout the development initiatives was to enhance the quality and transparency of assessment practices considering GAI capabilities. Understanding the end users, in this case, the students, is crucial and is known to facilitate successful implementation.^23,24^ A student survey was developed to explore GAI use and perceptions. The survey was distributed in the mid-spring semester of 2024, followed by a survey reminder. Participants obtained informed consent before taking part. At the end of the semester, the course directors completed a survey that included questions in adaptations implemented, planned adaptations for the next semester, and the perceived advantages, disadvantages and risks associated with the assessment tasks and learnings activities in relation to GAI usage.

## Results

### QIF Phase 3: Ongoing Structure Once Implementation Begins

Adaptations to assessment tasks and a standardized text on permitted GAI use were implemented in January 2024 across all courses. While the core principles were consistent, the specific adaptations varied by course to align with pedagogical goals. To increase transparency in the writing process, several strategies were introduced, including teacher checkpoints, peer reviews, and the submission of screenshots showing search history in academic databases. Additional measures included requiring students to anchor written assignments in personal patient cases or to incorporate specific course content, thereby reducing the risk of AI-generated submissions. To ensure the assessment accurately reflected students’ knowledge, alternative formats such as oral presentations (in seminars or audio recordings), application of knowledge to new cases, or responses to novel questions were implemented (Table 1). Evaluation of these implementations through structured reflections and feedback is a key component of phase 3. A central strategy was to preserve or enhance the assessment of CR and critical thinking. Examples of how GAI-related adaptations support the assessment of CR are illustrated in Table 2.

**Table 2.** Examples of GAI-Related Adaptations Supporting the Assessment of Clinical Reasoning.

Semester	Adaptation	Purpose and impact
5	Students receive patient cases and prepare for clinical exams in classrooms without internet access	Ensures independent hypothesis formulation and promotes clinical reflection on knowledge and knowledge gaps, minimizing reliance on GAI
6	Increased emphasis on oral elaboration of clinical reasoning during seminars and group discussions. Reduced length in some written assignments	Encourages interactive dialogue, deeper reasoning, and the ability to respond to questions based on relevant hypotheses and applied knowledge

Abbreviation: GAI, generative artificial intelligence.

Out of 224 physiotherapy students, 123 (55%) responded to the survey on GAI usage patterns and perceptions (Table 3). GAI usage varied widely, from 13% using it daily to 24% never using it. Forty-two percent felt they received sufficient information on permitted GAI use, and 49% found the study guide clear on what is permitted and prohibited. The survey included 4 questions measuring the frequency of GAI use for various academic purposes, using a Likert scale ranging from 1 (often) to 5 (never). Brainstorming ideas was the most common use of GAI, with only 29% of students never using it for this. Other features of GAI were seldom used, such as writing questions to oneself with 66% of students never using this. Most students, 61% and 76% respectively, reported never using GAI to rewrite or improve written texts for submission tasks or generate new texts for submissions. Further, in an open-ended question about other reasons for using GAI, the answers were grouped into (1) *Linguistically*: synonyms, translating, summaries, improving students’ texts; (2) *Helping in understanding*: explaining concepts, simplifying complex processes, for example, physiology or immunology, comparing differences in descriptions of complex processes, ensuring understanding by asking different questions, in self-studies, when a teacher cannot be asked; (3) *Making case scenarios;* and (4) *Searching literature*. Regarding students’ suggestions for learning support elements, the answers were grouped into (1) W*orkshops or written reports*: with scenarios and feedback on prompts and (2) *Introduction speeches*: about permitted use, prompting, and the importance of not using GAI to generate full texts.

**Table 3.** Mid-Semester Survey on Physiotherapy Students’ Experiences and Perceptions of GAI (Response Rate 123 Out of 224 Students (55%).

Survey questions	*n*	(%)
How often d you use GAI such as ChatGPT in your studies?		
Daily	16	(13)
Weekly	52	(43)
Monthly	28	(23)
Never	30	(24)
To what extent do you consider generative AI such as ChatGPT has contributed to your learning?		
Very high degree	8	(6)
High degree	44	(36)
Neither high nor low degree	31	(24)
Low degree	15	(12)
Not at all	25	(20)
Do you need a learning activity to be able to use generative AI such as ChatGPT optimally in your learning?		
Yes	21	(17)
Doubtful	53	(43)
No	52	(42)
To what degree do you think you received sufficient information about the guidelines for permitted and prohibited use of generative AI such as ChatGPT in the physiotherapist program?		
Very high degree	13	(11)
High degree	39	(32)
Neither high nor low degree	45	(37)
Low degree	22	(18)
Not at all	4	(3)
Do you find the text clear in the study guide about permitted and prohibited use of generative AI like ChatGPT in the physiotherapist program?		
Completely agree	14	(11)
Agree	46	(37)
Neither nor	35	(28)
Disagree	10	(8)
Do not agree at all	2	(2)
Have not read the study guide	16	(13)
How do you think your use of generative AI such as ChatGPT will change in the coming year?		
Reduced use	0	(0)
Slightly reduced use	1	(1)
Unchanged	55	(45)
Slightly increased use	52	(42)
Increased use	15	(12)

Abbreviation: GAI, generative artificial intelligence.

Teachers’ AI literacy was demonstrated during status updates and unit meetings, where they actively took ownership of the process by contributing to the integration of AI literacy into their courses and taking responsibility for implementing necessary pedagogical adjustments. In the course director survey, their reflections on course assessment tasks revealed an awareness of which assessment tasks remained vulnerable to GAI misuse. They also proposed further adaptations to improve both assessment tasks and learning activities.

### QIF Phase 4: Improving Future Applications

The student survey indicated a need for more information and support on using GAI, along with valuable suggestions for teaching and learning activities. The course directors’ survey at the end of the semester resulted in further course adaptation initiatives, focusing on educational strategies to prevent misuse of GAI. These included integrating empirical data, allowing structured use of GAI, and enhancing AI literacy (Table 1). Since the autumn semester of 2024, learning activities, assessment tasks and learning outcomes related to GAI have gradually been implemented in semesters 1 to 3. These components are aligned with the principles of PBL, using case-based problem solving, peer collaboration, and structured feedback from both teachers and librarians (Table 4).

**Table 4.** Integration of GAI into PBL to Foster Reflective and Critical AI Literacy.

Semester	New learning activity/assessment task	Description
1	Introduction to GAI	Focus on basic prompting, data security and ethical considerations, led by a librarian. Students engage in small-scale problem-solving cases and receive formative feedback
2	GAI-supported information retrieval	Builds on prior knowledge, introduction of Scopus AI for database searches. A preparatory learning activity supports an assessment task involving the search and presentation of intervention study results
3	Critical review of AI-generated content	Students use GAI to answer specific questions, evaluate the reliability of the responses, and discuss findings in groups. In a seminar, co-led by a teacher and a librarian, students present the results, discuss how the information was verified, and which sources they used

Abbreviation: GAI, generative artificial intelligence.

In course evaluations, 2 specific questions address students’ perceptions of the information provided on GAI. To remain aligned with the rapid advancements in the field, a 1-year follow-up student survey is planned. Insights gathered from student-teacher forums, end-of-semester course evaluations and the student survey collectively form a robust foundation for continued development of pedagogical strategies.

## Discussion

Our structured approach involved the faculty's collaborative efforts, implementing pedagogical adaptations in a physiotherapy program and single-subject courses. Assessment tasks at risk of AI-generated submissions were identified and adapted to mitigate this risk, with a focus on preserving CR essential for future healthcare roles. The development initiatives challenged the faculty's theoretical knowledge and professional judgment about GAI. Insights from status update meetings, unit meetings and the course directors’ survey indicated a growing AI literacy among teachers, reflected in their sense of responsibility and creativity in implementing and planning new course adaptations. The faculty's engagement with PBL facilitated a culture of solution-focused practices,^25^ which supported active participation and informed decision making during the GAI initiatives.

Constructive alignment^22^ contributed to efficient group work creating adaptations to assessment tasks aligning with desired learning outcomes. The QIF^13^ supported the structure and promoted the efficient use of resources. Key factors enabling impactful course adaptations within 4 months included structure and planning, engaging experts, collegial culture, and strong pedagogical leadership, elaborated upon in the following sections.

Our objectives were first clarified, followed by the creation and adherence to a structured plan. The use of frameworks, such as the QIF framework,^13^ is recommended to guide implementation processes^26^ and it provided structure, helped identify needs, resources, and supported strategies for successful implementation. Participation by the entire faculty in the workshop was essential for a common understanding of GAI challenges and facilitated the whole work process.

Early time planning, and precise task assignments are necessary to ensure efficient use of staff time. Three team members with diverse skills reviewed all assessment tasks in the courses, ensured consistent judgments as concerns constructive alignment and GAI misuse risks. Constructive alignment is a successful strategy for working with course and curriculum changes to ensure that teaching and learning activities and assessment tasks support the students in reaching the intended learning outcomes.^27^ In our work, constructive alignment, together with the digital matrix, was crucial in highlighting the assessment tasks that needed to be addressed and adapted for the use of GAI. Additionally, teachers familiarized themselves with GAI, the university's guide and recommendations for the use of GAI, likely improved efficiency at the workshop. This approach resembles students’ preparation before teaching activities,^28^ to enhance motivation, improve discussions and deepen knowledge.

Combining our unit's resources with the expertise of GAI, we not only enhanced our learning but also fostered ongoing collaboration. We have now engaged another expert unit, the university library, in our introduction in GAI in semesters 1 to 3, thereby increasing our collaboration around GAI.

A strong collegial culture within the faculty supported effective development. Our Community of Practice,^29^ defined by a shared profession, as both physiotherapists and teachers and the skills of PBL-experienced teachers was an important resource. PBL methodology enhances social abilities, problem-solving capabilities, self-directed learning, and communication skills.^25^ Within our group, these skills have likely facilitated effective teamwork and streamlined processes. Strong pedagogical leadership, with both formal and informal mandates from the group, further strengthened the process. All these factors, a collegial culture, a clear direction for the development initiatives, and strong pedagogical leadership^30,31^ are factors known to drive successful educational development.

### Educational Strategies for Ethical and Informed GAI Engagement

Recent course adaptations involving GAI have been shaped by principles from both PBL^14,15^ and clinical reasoning.^6^ These approaches share a focus on authentic problem-solving, hypothesis generation, and critical analysis of knowledge gaps^7,16^—skills essential for both academic and clinical reasoning. The implemented educational strategy emphasizes the learning process rather than just the final product. For example, reviewing drafts or incorporating peer review before submission helps identify AI-generated content while also facilitating deeper understanding and feedback skills^32^—also core elements of PBL.^16^ Similarly, requiring students to submit audio reflections or apply knowledge in case-based scenarios enhances assessment of comprehension and supports the development of clinical reasoning. Seminars with student presentations further promote applied knowledge, offer opportunities for feedback, and reduce plagiarism risks. In some cases, written assignments are shortened to allow more time for these discussions, though this may limit writing practice.

To support both students and faculty, a standardized text on permitted GAI use has been introduced. This helps guide classroom discussions on academic integrity and encourages responsible use of AI tools. Together, these adaptations reflect a shift toward more reflective, collaborative, and ethically grounded learning environments.

### Activities to Enhance AI Literacy

To enhance teachers’ AI literacy, targeted activities were implemented, aligning with Sperling et al’s recommendation to address GAI in theoretical, practical, and ethical contexts.^33^ The faculties’ course adaptations plans reflected an improved understanding of GAI. However, it is crucial to consider whether sufficient support is provided for teachers to build confidence and trust in future GAI teaching. Trust is essential in student-teacher interactions, with cognitive competence being a key component. Other important aspects of trust include showing interpersonal care, sensitivity to own and other identities and adherence to principles, that is, educational, professional, and cultural.^34^ Our approach to supporting AI literacy includes structural planning, collaborative group work, and feedback on theoretical, practical and ethical aspects. Continued collaboration with GAI experts and the university library further reinforces this development. These efforts aim not only to deepen understanding but also to promote responsible and confident use of GAI in educational settings. The rapid advancement of AI significantly impacts the conditions for higher education, underscoring the necessity for faculty to stay abreast of these developments.

### Student use of GAI and Implications for Teaching and Learning Design

The student survey revealed diverse uses of GAI in academic work, along with constructive suggestions for teaching and learning activities. While co-designing learning experiences with students—recommended in AI-enhanced education^35^—has not yet been implemented, the survey findings, together with student-teacher interactions and course evaluations, have informed current adaptations. These insights will continue to guide the development of targeted GAI-related learning and teaching activities.

In the current survey, only 24% of the students reported that they had never used GAI in their studies, compared to 48% in a similar study among medical students 1 year earlier.^36^ This increase highlights the rapid adoption of GAI and underscores the urgency of adapting learning activities and assessment tasks accordingly.

Brainstorming ideas emerged as the most common use of GAI in both our survey, and in previous research among medical students,^10^ also described as commonly used in our focus group study on students.^5^ This is particularly noteworthy, as brainstorming and problem-solving are central in PBL^25^ and are also key components of clinical reasoning.^7^ The alignment between students’ natural use of GAI and core educational strategies suggests a valuable opportunity to integrate AI tools in ways that reinforce essential academic and clinical reasoning skills.

The significant variation in students’ use of GAI suggests a need for more information and learning activities. Previous reports indicate that students’ perceptions of risks related to plagiarism, cheating, attitudes toward technology, and perceived usefulness are key factors influencing their use of GAI.^37^ Additionally, there may be a disparity between students who pay for advanced ChatGPT and those who do not.^2^ To address these variations, we plan to provide more information, utilize student-teacher forums, and incorporate GAI-focused learning activities.

### Methodological Considerations

When interpreting the student survey results it should be noted that the questions were not validated, and the response rate was 55%. Another limitation is the lack of structured evaluation of AI literacy among teachers. A recommendation for future research is to use validated assessments to enhance understanding of teachers’ AI literacy. The GAI initiative was conducted in a physiotherapy program using a PBL approach, although the structure is likely transferable to other courses within the Faculty of Medicine and Health Sciences.

## Conclusion

The systematic approach using QIF and PBL, expert support, faculty champions, problem-solving strategies, and feedback, enabled meaningful curricular changes within 4 months. The variability in student GAI use underscores the need for equitable AI literacy education. This approach not only reduced the risk of GAI misuse but also enhanced faculty preparedness, offering a scalable model for other health sciences programs.

## Supplemental Material

sj-docx-1-mde-10.1177_23821205251378794 - Supplemental material for Structured Development of Learning and Assessment Tasks to Prevent Generative AI Misuse and Enhance AI Literacy in the Faculty in Physiotherapy Education Supplemental material, sj-docx-1-mde-10.1177_23821205251378794 for Structured Development of Learning and Assessment Tasks to Prevent Generative AI Misuse and Enhance AI Literacy in the Faculty in Physiotherapy Education by Yvonne Lindbäck, Karin Valeskog, Karin Schröder and Sofi Sonesson in Journal of Medical Education and Curricular Development
