# Supplementary material for: Structured Development of Learning and Assessment Tasks to Prevent Generative AI Misuse and Enhance AI Literacy in the Faculty in Physiotherapy Education
Source: J Med Educ Curric Dev. 2025 Sep 16;12:23821205251378794. doi: 10.1177/23821205251378794 (PMC12441287; doi:10.1177/23821205251378794)
Supplement: sj-docx-1-mde-10.1177_23821205251378794 - Supplemental material for Structured Development of Learning and Assessment Tasks to Prevent Generative AI Misuse and Enhance AI Literacy in the Faculty in Physiotherapy Education [file sj-docx-1-mde-10.1177_23821205251378794.docx]

Standards for QUality Improvement Reporting Excellence in Education: SQUIRE-EDU

STRUCTURED DEVELOPMENT OF LEARNING AND ASSESSMENT TASKS TO PREVENT GENERATIVE AI MISUSE AND ENHANCE AI LITERACY IN PHYSIOTERAPY EDUCATION

Citation: Ogrinc G, Armstrong GE, Dolansky MA, Singh MK, Davies L. [SQuIRE-EDU (Standards for QUality Improvement Reporting Excellence in Education0: publication guidelines for educational improvement. Academic Medicine. 2019 Oct 1;94(10):1461-70.](https://www.squire-statement.org/document/docWindow.cfm?fuseaction=document.viewDocument&ID=B895CE8E2D7255CD149F01E624B8CA205C608AE0404F1E537A5DF2F1E483E65F72D222CF3C06BF07C605AF2A49135A43)

| **Text section and item name** | **SQUIRE item description** | **SQUIRE-EDU extension description** | **Page in the manuscript “Structured development of learning and assessment tasks to prevent generative AI misuse and enhance AI literacy in physioterapy education”** |
| --- | --- | --- | --- |
| Notes to authors | The SQUIRE guidelines provide a framework for  reporting new knowledge about how to improve healthcare.  The SQUIRE guidelines are intended for reports that describe system level work to improve the quality,  safety, and value of healthcare, and used methods to establish that observed outcomes were due to the  intervention(s).  A range of approaches exists for improving healthcare. SQUIRE may be adapted for reporting any of these.  Authors should consider every SQUIRE item, but it may be inappropriate or unnecessary to include every SQUIRE element in a particular manuscript.  The SQUIRE glossary contains definitions of many of the key words in SQUIRE.  The Explanation and Elaboration document provides specific examples of well-written SQUIRE items, and an in-depth explanation of each item.  Please cite SQUIRE when it is used to write a manuscript. | The SQUIRE-EDU extension of the SQUIRE guidelines provides  a framework intended to increase the completeness,  transparency, and replicability of published reports that describe  systematic efforts to improve health professions education.  They apply to all learning settings (e.g., classroom, simulation, clinical, etc.). The guidelines encourage the description of the process and context of educational change, use of iterative cycles, and use of data over time.  Authors should consider every SQUIRE and SQUIRE-EDU item,  but it may be inappropriate or unnecessary to include every SQUIRE and SQUIRE-EDU element in a particular manuscript.  Not all items have an EDU extension. If there is no EDU extension, use the SQUIRE item. If there is an EDU extension, it  may be used on its own or in conjunction with the SQUIRE item.  Educators use a range of systematic methods to make  education and healthcare demonstrably better. SQUIRE-EDU may be adapted for reporting any of these methods.  Please cite SQUIRE-EDU when it is used to write a manuscript. |  |

| **Title and abstract** | | | |
| --- | --- | --- | --- |
| **Text section and item name** | **SQUIRE item description** | **SQUIRE-EDU extension description** | **Headline in the manuscript “Structured development of learning and assessment tasks to prevent generative AI misuse and enhance AI literacy in physioterapy education”** |
| 1. Title |  | EDU 1: Indicate that the manuscript concerns efforts to improve health professions education systems and learning | Title page |
| 1. Abstract |  | EDU 2: Keywords include a focus on education and learning | Title page, abstract |
| **Introduction: Why did you start?** | | | |
| 1. Problem description |  | EDU 3: Description of the nature and significance of the need for change in the local educational system | Introduction |
| 1. Available knowledge | Summary of what is currently known about the problem, including relevant previous studies | - | Introduction |
| 1. Rationale |  | EDU 5: Identify the guiding theory (learning, change,  implementation, or other) and how it aligns with the need for change in the local educational system | Introduction |
| 1. Specific aims | Purpose of the project and of this report | - | Introduction |
| **Methods: What did you do?** | | | |
| 1. Context |  | EDU 7a: Contextual elements for learning (e.g., setting, program, people, resources, social, geopolitical influences)  before the intervention(s)  EDU 7b: The interrelationships between the contextual elements and the local educational and healthcare systems before the intervention(s) | Method  Method; QIF phase 1: Initial considerations |
| 8. Intervention(s) |  | EDU 8a: Description of the primary interventions and cointerventions (e.g., faculty or tool development)  EDU 8b: Specify how the interprofessional education team (e.g., faculty, staff, patients, and learners) was part of the design of the intervention | Method; QIF phase 2: Creating a structure for implementation |

| **Text section and item name** | **SQUIRE item description** | **SQUIRE-EDU extension description** | **Headline in the manuscript “Structured development of learning and assessment tasks to prevent generative AI misuse and enhance AI literacy in physioterapy education”** |
| --- | --- | --- | --- |
| 9. Study of the  intervention(s) |  | EDU 9a: Approach used to understand the impact of the  educational intervention(s) on the learner and beyond, such as impact on patients, families, the community, faculty, educational program, or the healthcare system  EDU 9b: Approach to assess the fidelity of and the iterative changes to the planned intervention(s) over time | Method; QIF phase 2: Creating a structure for implementation |
| 10. Measures |  | EDU 10: Quantitative and/or qualitative measures chosen to assess the educational processes and outcomes on learners,  faculty, educational programs, patients, families, healthcare systems, or communities | Method; QIF phase 2: Creating a structure for implementation |
| 11. Analysis | a. Qualitative and quantitative methods used to draw inferences from the data  b. Methods for understanding variation within the data, including the effects of time as a variable | - | Method; QIF phase 2: Creating a structure for implementation  11b; Not applicable |
| 12. Ethical considerations |  | EDU 12: Approaches to address vulnerability of learner participants | Statements and declarations;  Ethical considerations |
| 13. Results: What did you find? | b. Details of the process measures and outcome  c. Contextual elements that interacted with the  intervention(s)  d. Observed associations between outcomes, interventions, and relevant contextual elements  e. Unintended consequences such as unexpected  benefits, problems, failures, or costs associated with the intervention(s)  f. Details about missing data | EDU 13a: For each educational intervention and cointervention, provide details about iterative modifications  based on the assessment of the learning | Results;QIF phase 3: Ongoing structure once implementation begins Table 1 and 2 QIF phase 4: Improving future applications13f; QIF phase 3: Ongoing structure once implementation begins, information, but no further analysis of missing data;*“Out of 224 physiotherapy students, 123 (55%) responded to the survey on GAI usage patterns and perceptions*” |

| **Text section and item name** | **SQUIRE item description** | **SQUIRE-EDU extension description** | **Headline in the manuscript “Structured development of learning and assessment tasks to prevent generative AI misuse and enhance AI literacy in physioterapy education”** |
| --- | --- | --- | --- |
| **Discussion: What does it mean?** | | | |
| 14. Summary |  | EDU 14: Connect the findings to the guiding theory (learning, change, implementation, other) used to direct the change in the local educational system | Discussion |
| 15. Interpretation | a. Nature of the association between the  intervention(s) and the outcomes  b. Comparison of results with findings from other publications  d. Reasons for any differences between observed and  anticipated outcomes, including the influence of context  e. Costs and strategic trade-offs, including  opportunity costs | EDU 15c: Include the impact of the intervention(s) on  learners, faculty, educational program, patients, families,  healthcare systems, or communities | Discussion |
| 16. Limitations | a. Limits to the generalizability of the work  b. Factors that might have limited internal validity such as confounding, bias, or imprecision in the  design, methods, measurement, or analysis  c. Efforts made to minimize and adjust for limitations | - | Discussion; Methodological considerations  16c. For example, in *Method QIF phase 2: Creating a structure for implementation*: A survey reminder was used to minimize the limitation with low response rate. |
| 17. Conclusions | a. Usefulness of the work  c. Potential for spread to other contexts  e. Suggested next steps | EDU 17b: Scalability of the work to other learners and  contexts  EDU 17d: Lessons learned for clinical practice, education, and policy | Conclusion |
| **Other information** | | | |
| 18. Funding | Sources of funding that supported this work. Role, if any, of the funding organization in the design, implementation, interpretation, and reporting | - | Funding |
